# The application of encapsulation technology in the food Industry: Classifications, recent Advances, and perspectives

**DOI:** 10.1016/j.fochx.2024.101240

**Published:** 2024-02-19

**Authors:** Yaguang Xu, Xinxin Yan, Haibo Zheng, Jingjun Li, Xiaowei Wu, Jingjing Xu, Zongyuan Zhen, Chuanlai Du

**Affiliations:** a College of Food Engineering, Anhui Science and Technology University, Chuzhou 233100, China; b The Institute of Functional Agriculture (Food) Science and Technology at Yangtze River Delta (iFAST), Chuzhou 239000, China; c Anhui Provincial Key Laboratory of Functional Agriculture and Functional Food, Chuzhou 233100, China

**Keywords:** Encapsulation, Antioxidation, Delivery, Probiotics, Stability

## Abstract

Encapsulation technology has been extensively used to enhance the stability, specificity, and bioavailability of essential food ingredients. Additionally, it plays a vital role in improving product quality and reducing production costs. This study presents a comprehensive classification of encapsulation techniques based on the state of different cores (solid, liquid, and gaseous) and offers a detailed description and analysis of these encapsulation methods. Specifically, it introduces the diverse applications of encapsulation technology in food, encompassing areas such as antioxidant, protein activity, physical stability, controlled release, delivery, antibacterial, and probiotics. The potential impact of encapsulation technology is expected to make encapsulation technology a major process and research hotspot in the food industry. Future research directions include applications of encapsulation for enzymes, microencapsulation of biosensors, and novel technologies such as self-assembly. This study provides a valuable theoretical reference for the in-depth research and wide application of encapsulation technology in the food industry.

## Highlights

• A comparison of encapsulation technologies for various core materials.
• Versatile applications and functions of encapsulation in the food industry.
• Potential impacts of encapsulation on food processing and preservation.
• Future in-depth research and novel approaches of encapsulation technology.

## Graphical abstract

**Figure:**
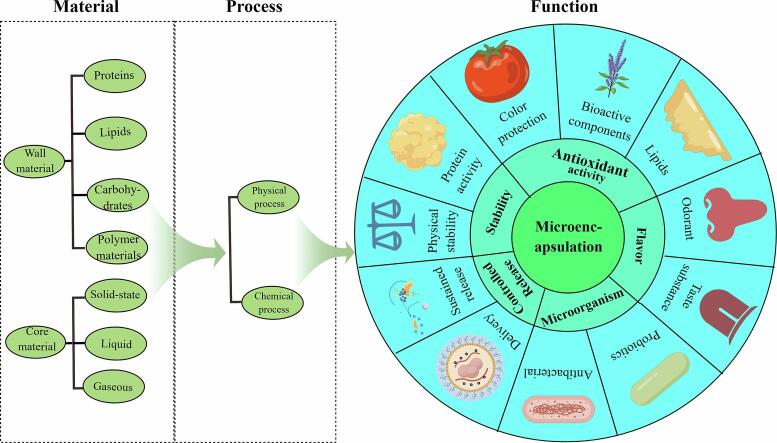

## 1 Introduction

Encapsulation technology originated in the 1950 s during the exploration of pressure-sensitive wall materials for the production of carbonless copy paper (Green & Scheicher, 2007). Since then, this technology has found widespread applications in various industries, including chemical, casting, medical, and pharmaceutical. In the 1970 s, the application of encapsulation technology expanded into the food industry (Calderón-Oliver & Ponce-Alquicira, 2022), initially focusing on food additives, ingredients, health foods, and probiotics. However, as research expanded and technology evolved, the capabilities of encapsulation technology have diversified greatly, offering various functions in food processing. The main functions of encapsulation technology are shown in Table 1. The main applied technologies for encapsulation in foodstuffs include spray–drying, extrusion, freeze–drying, coagulation, and adsorption techniques (Yan et al., 2022). Recently, various encapsulation techniques have emerged, especially with the application of different core and wall materials, which have greatly enriched the potential of encapsulation technology. Currently, encapsulation technology plays a crucial role in addressing three significant challenges in the food industry. First, it addresses the issue of food ingredient instability, which encompasses problems like protein and lipid oxidation, as well as the deterioration of active ingredients and pigment fading or discoloration. Second, it facilitates the controlled release of food ingredients, enabling sustained release and efficient delivery of additives, probiotics, and other essential ingredients. Third, it enhances the sensory quality of food by either adsorbing or masking taste and odor substances. This study provides an in-depth review of the recent applications of encapsulation technology in food processing. It delves into various aspects, including the principles of encapsulation technology, the classification of core materials, and the underlying principles, effects, and influencing factors of encapsulation technology in achieving various functions in food processing.

**Table 1:** Main features of the encapsulation technique.

Function	Content
Pulverization (George et al., 2021)	Solidify gas and liquid raw materials that are difficult to process and store to improve their solubility, fluidity, and storage stability.
Reduce volatility (Gu et al., 2023)	Prevents volatilization of flavor components and reduces flavor loss.
Decreased virulence (Acar, 2021)	Reduces the toxicological effects of food additives and other substances.
Improve the stability of the material (Sarabandi & Jafari, 2020)	Many food encapsulation treatments can prevent oxidation and protect against ultraviolet, temperature, and humidity effects. This ensures the preservation of nutrients and special functions.
The incompatible components can be mixed uniformly (Li et al., 2019)	Encapsulation techniques enable components that may react with each other to be embedded separately, ensuring their stability within the system. This allows various effective components to be released in an orderly manner, exerting their effects at corresponding times, thus enhancing the flavor and nutrition of food products.
Taste masking (Gao et al., 2022)	Unpleasant tastes or odors of certain nutrients can be masked through encapsulation techniques. The encapsulated product does not dissolve in the oral cavity but releases its contents in the digestive tract for nutritional benefits.
Isolated active ingredients (Chen et al., 2020)	Encapsulation preserves the active effect of micro-nutrients and physiologically active substances in food, ensuring their beneficial impact on the human body.
Controlled release (Yue et al., 2020)	The embedded material provides a specific release mode through a pre-designed dissolution and release mechanism. This allows precise control over the timing and quantity of release and action.

## 2 Overview

Encapsulation technology is a collective term for technologies that use encapsulation to enclose certain substances, aiming to improve their solubility, polarity, and other properties or enhance their stability to external factors such as light, heat, and oxidation. The substance being encapsulated is called the core material, while the material used for encapsulation is called the wall material (Yan et al., 2022). In food processing, the role of the wall material is multifaceted. It helps to protect the core material from various environmental factors, including oxygen, temperature, moisture, and ultraviolet radiation. Moreover, it contributes to enhancing product stability and prolonging shelf life. Additionally, it plays a pivotal role in improving the physical properties of the core materials, such as their solubility, hydrophilicity, and lipophilicity. Furthermore, the wall material controls the release conditions and rate of the core material, ensuring precise delivery. It also protects against any unpleasant taste and odors associated with the core material, thereby improving overall product quality (Bu et al., 2021). Encapsulation processes can be categorized into chemical and physical technologies (see Table 2). The encapsulation rate, release characteristics, and efficacy changes are often used as important indicators to evaluate the effectiveness of encapsulation (Kotliarevski et al., 2021).

**Table 2:** Main encapsulation process performance and classification (Sousa et al., 2022).

Technology	Encapsulation method	Particle size/µm	Maximum encapsulation rate/%
	Simple condensates	20–200	＜60
Chemical Process	Complex coacervation	5–200	70–90
	Molecule contains	5–50	5–10
	Spray–drying	1–50	＜40
Physical Process	Spray–freezing	20–200	10–20
	Extrusion	200–2000	6–20
	Fluidized bed	＞100	60–90

### 2.1 Classification

Encapsulation techniques can be classified into several categories based on their carrier material and methods used, such as gel encapsulation (grid-type encapsulation) and semi-permeable membrane encapsulation (microcapsule encapsulation) (Jamuna & Ramakrishna, 1992). Based on different types of delivery systems, they are further divided into liposomes, nanoparticles, emulsions, and microcapsules (Kothale et al., 2020). Additionally, based on the state of the core material, encapsulation techniques are categorized into solid, liquid, and gaseous states. Each state of core material requires specific encapsulation techniques (see Table 3).

**Table 3:** Classification and application technology of encapsulated core materials (Yuan et al., 2023).

Core Material State	Applied technology
Solid	Spray–drying technology, spray–cooling–drying, fluidized bed, oil-phase separating method, extrusion, interfacial polymerization, air suspension, and electrostatic spinning.
Liquid	Composite phases emulsion method, molecular encapsulation method, tiny hole-coagulation method, liposome encapsulation technology, emulsion encapsulation, complex coacervation, and nanoencapsulation technology.
Gaseous	Supercritical impinging stream technology.

### 2.2 Principles

The encapsulation process generally involves two steps: first, emulsification of the core material (e.g., “lipid-essence” system) with a certain density of wall material solution (e.g., polysaccharide or protein solution); second, drying or condensing the emulsion (Zhao et al., 2019). The choice of encapsulation method can result in different shapes and structures of the encapsulation substrate, which may affect the diffusion of the encapsulated or external substances (e.g., oxygen and solvents) and their stability in food (Green & Scheicher, 2007). Food core materials include esters, ethers, ketones, alcohols, water, glycerin, menthol, flavors, pigments, enzymes, vitamins, amino acids, oils, fats, condiments, food additives, and probiotics. When using encapsulation technology for food products, it is crucial to consider both the encapsulation rate and cost of production. Based on the core material, different wall materials and encapsulation techniques are selected to achieve maximum encapsulation rate at a lower cost, suitable for processing and production. The core materials are classified into solid, liquid, and gaseous states.

#### 2.2.1 Solid-state core materials

Solid-state core materials have small inter-particle distances and high forces, maintain a certain volume and shape, and exhibit poor fluidity. Generally, they lack free-moving ions, and the nature of their conductivity is usually due to the number of free electrons. When subjected to external forces, their volume and shape change slightly. Encapsulation treatment of solid-state core materials can alter their solubility, hydrophilicity, and hydrophobicity, making them easier to digest and absorb in the human body and convenient to consume, which enhances taste and flavor, as well as extends shelf life (Luo et al., 2022). For example, microencapsulation of solid-state core material substances for making instant beverages enhances the quality and value of beverages and enriches the taste and flavor (Kim et al., 2019). Table 4 presents an overview of the present encapsulation technology applied to solid-state core materials, along with its methods, principles, and characteristics.

**Table 4:** Technical principles and characteristics of solid/liquie/gas-state core encapsulation systems.

	Encapsulation system	Method/Principle	Features
**Solid-state core encapsulation systems**	Spray–Drying Technology (Wong et al., 2020)	The prepared emulsion is dried into granules through atomization in a hot air stream of the spray unit, followed by mass and heat exchange.	Advantages: short drying process, good solubility, low cost, convenient transportation and storage, and easy process operation. Disadvantages: uneven particle size and partial rupture of the core material on the surface is easily oxidized.
	Spray–Cooling–Drying (Guo et al., 2020)	The core material is combined with the emulsifier and wall material to form a suspension emulsion. Microcapsules are created by freezing at a temperature of − 20 °C and then placed in a freeze dryer through the physical change of water (sublimation).	Advantages: minimal core material damage. Disadvantages: dried powder requires crushing and sieving, and equipment requirements are higher.
	Fluidized Bed (Frank et al., 2023)	The hot air-flow of the fluidized bed is used to wrap the core material with the wall material solution to create microcapsules.	Advantage: uniform and moderate wall thickness, which is conducive to large-scale production. Disadvantages: easy to damage the surface and low yield.
	Oil-Phase Separating Method (Zhang et al., 2023)	The core material is added to the solution containing the shell material polymer and solvent, mixed, and dispersed into a suspension. Then, a nonsolvent liquid to the polymer and a precipitation solvent is added to initiate phase separation, encapsulating the core material into microcapsules.	Advantages: simple equipment and a wide range of shell material sources. Disadvantages: some solvents lead to potential environmental pollution and higher risks.
	Extrusion (Hu et al., 2021)	Commonly used pore membrane extrusion method, where the emulsion formed by the core material and the wall material is extruded through the pore membrane at low temperatures. When the wall material comes into direct contact with the dehydrating agent, microcapsules are prepared due to dehydration.	Advantages: better closure of the capsule wall membrane and minimal chances of flavor substances being affected. Disadvantages: low surface oil content, resulting in a long storage period and a lower yield.
	Interfacial Polymerization (Ricardo et al., 2021)	Two monomers with different solubility are uniformly added to the continuous phase of the wall material and the dispersed phase of the core material. A polycondensation reaction occurs at the phase interface, resulting in the formation of microcapsules.	Advantages: good densification and faster reaction rates. Disadvantage: some of the monomers may be retained in the microcapsules.
	Air Suspension (Sun et al., 2022)	The aqueous solution containing the wall material is sprayed on the surface of the suspended core material particles to increase their mass. Then, the solvent is vaporized using low hot air, causing the particles to rise again due to the reduced mass. This process can be repeated to obtain solid particles.	Advantages: a wide range of optional wall materials, and the thickness of the capsule wall after molding is uniform and moderate. Disadvantages: more control factors and limitations regarding the core material.
	Electrostatic Spinning (Fan et al., 2021)	Make the charged polymer solution (or melt) flow deform in an electrostatic field, followed by solvent evaporation or melt–cooling and curing to obtain fiber-like material.	Advantages: simple, easy to operate, no organic solvents, low cost, high production efficiency. Disadvantages: difficult to obtain nanofiber filaments or separate staple fibers, resulting in very low yield and strength in the electrostatic spinning process.
**Liquid core encapsulation system**	Phases Emulsion Method (Du et al., 2022)	Core material is first combined with the emulsifier, then mixed with the wall material to form a composite emulsion. The microcapsules are obtained by curing after removing the solvent in the suspension emulsion through evaporation or extraction.	Advantages: simple process, high emulsion stability, and infiltration prevention of the inner water phase core material. Disadvantages: residual toxic organic solvents.
	Molecular Encapsulation Method (Macetti & Genoni, 2019)	Technology for encapsulation of hydrophobic core using a hollow and hydrophobic internal structure carrier.	Advantages: long-lasting storage, easy preparation without requiring special equipment. Disadvantages: requires the same core diameter, resulting in low product load.
	Tiny Hole-Coagulation Method (Liu et al., 2019)	Microcapsules are formed by placing a mixture of core and wall materials into a tiny hole-coagulation device and adding chemicals such as calcium chloride or aldehydes for a cross-linking reaction.	Advantages: simple operation and no use of organic solvents. Disadvantages: larger particle size and low encapsulation rate.
	Liposome Encapsulation Technology (Zhao et al., 2022)	Wall material is a spherical or approximately spherical vesicle with a biofilm structure, typically consisting of one or more phospholipid bilayers or thin layers.	Advantages: effectively contains molecules in a phospholipid bilayer, reducing degradation in extreme environments. Disadvantages: difficult removal of organic solvents and synthetic surfactants, demanding storage conditions.
	Emulsion Encapsulation (Bago Rodriguez & Binks, 2022)	In the oil–water system, an emulsion is formed by adding appropriate surfactants with strong mixing, creating colloidal particles to be encapsulated, thereby protecting the core material.	Advantages: simple preparation process, improved digestibility, antibacterial activity, and antioxidant activity. Disadvantages: low physical stability and prone to demulsification in extreme environments.
	Complex Coacervation (Muhoza et al., 2022)	After dilution, pH value, or temperature adjustment, the reaction between the wall materials is condensed and precipitated, and finally, the microcapsules are prepared, resulting in the preparation of microcapsules.	Advantages: mild preparation process, high efficiency, and low impact on biological activity. Disadvantages: difficult control of reaction conditions and production costs.
	Nanoencapsulation Technology (Yin et al., 2020)	Bioactive substances are encapsulated in the nanoscale through nanocomposite, nanoemulsification, and nanoconstruction. Nanocapsules can release their contents at a controlled rate.	Advantages: higher stability, improved *in vivo* absorption, effective core quality improvement, safety, and functionality. Disadvantages: requires high precision and is still in the laboratory stage.
**gaseous core material**	Supercritical Impinging Stream Technology (Méndez Cañellas et al., 2023);	The solute is dissolved in a supercritical fluid to saturate and then introduced into the low-pressure chamber through the preheating nozzle. The sudden decrease in pressure causes the solute to quickly reach saturation and liquefy, resulting in precipitation of very small particles. This process ensures a uniform particle size distribution due to instantaneous pressure changes in fluids.	Advantages: low-temperature process with minimal residual solvent in particles; solvent and antisolvent can be recycled, reducing organic solvent usage and environmental pollution; uniform particle size distribution with no significant molecular structure change and good morphology. Disadvantages: potential issues with nozzle antiblocking, sealing simplification, and high equipment investment.

#### 2.2.2 Liquid core material

Liquid core materials exhibit strong fluidity and can reach an equilibrium state under the action of pressure. However, it is susceptible to deformation when subjected to external forces such as tension or shear. Upon encapsulation, the liquid core material offers several advantages, including high stability, long-lasting storage, simple operation, fast reaction speed, and reduced degradation in extreme environments. Common encapsulation methods include the complex phase emulsion method, liposome encapsulation, and emulsion encapsulation (Damerau et al., 2022). Table 4 presents the current encapsulation technology applied to the liquid core material, along with their method, principle, and characteristics.

#### 2.2.3 Gaseous core material

Gaseous core materials exhibit strong fluidity, minimal inter-particle forces, and easy compressibility. When conventionally added to products, it tends to volatilize quickly, making it challenging to process and ensure stable quality. However, the controlled release of gaseous core material can be achieved through encapsulation treatment (Li et al., 2022). This encapsulation treatment is characterized by its preparation process, high encapsulation efficiency, less pollution, and minimal effect on biological activity. Table 4 presents the current encapsulation technology, methods, principles, and characteristics applied to gaseous core materials.

## 3 Application of encapsulation technology in foodstuffs

Encapsulation technology has the ability to alter the state of materials, isolate interactions between materials, reduce volatility, and control release. It was initially applied in medicine and healthcare products, particularly for functional ingredients, probiotics, etc. Over time, encapsulation technology has found significant application in the food industry (Boyano-Orozco et al., 2020), especially in areas such as antioxidation, stability, controlled release, microorganisms, and flavor.

### 3.1 Antioxidant activity

Oxidation in food not only leads to oil rancidity, fading, discoloration, and vitamin loss but also affects the sensory quality and nutritional value and may even produce harmful substances posing health hazards. Antioxidants in food act by effectively inhibiting free radicals or oxygen-related chemical reactions. Their mechanism of action can involve direct interaction with free radicals to prevent further reactions or consume oxygen in the environment. Adding antioxidants is a common approach to food antioxidation, and factors like thermal stability and efficiency impact their effectiveness in food applications (Poljsak et al., 2021). Encapsulation technology addresses the challenges of direct addition by isolating the core material from the external environment, preventing undesirable factors that could lead to oxidation during processing, thereby improving stability (Wang et al., 2023). For instance, several well-known infant formula brands, such as Wyeth, incorporate Docosahexaenoic Acid (DHA) and Eicosapentaenoic Acid (EPA), which are beneficial for the cognitive and visual development of infants. These essential fatty acids are introduced using techniques like spray–drying, which encapsulates fish oil into microcapsules. This not only protects polyunsaturated fatty acids (DHA, EPA) in fish oil from oxidation but also enhances their solubility and masks any undesirable odors (Solomando et al., 2020). In addition, encapsulation technology can enhance the synergistic effect between antioxidants and metal–ion chelating agents, significantly improving antioxidant capacity (Taktak et al., 2019). Specifically, the antioxidant activity of encapsulation technology is mainly applied to protect lipids, bioactive components, and pigments in food.

#### 3.1.1 Lipids

Lipid oxidation is a common form of food deterioration in products containing fats and oils, leading to undesirable changes in flavor, discoloration, and decreased nutritional value. Additionally, harmful substances may be produced. Encapsulation treatment offers a solution by encapsulating lipid molecules with inert substances, effectively preventing oxidation reactions and delaying the oxidation process. Encapsulation also helps to mask unwanted odors, improve product quality and safety, enhance nutritional value, and extend the shelf-life of foods (Le Priol et al., 2022). The choice of wall material plays a crucial role in the oxidation resistance of encapsulation lipids. When dealing with lipophilic core materials, hydrophobic wall materials are preferred for encapsulation. Commonly used wall materials for lipid encapsulation include carbohydrates, gums, and proteins. Amphiphilic proteins exhibit excellent emulsifying properties, reducing the surface tension between the oil and water phases to form a stable water-in-oil or oil-in-water system (Krstonošić et al, 2020). The use of mixed wall materials often yields better oxidation resistance compared to single-wall materials. Microcapsules prepared from a single-wall material are less stable, exhibit lower oxidation resistance, and have reduced polymerizability. The combination of different wall materials helps enhance mechanical properties (Damerau et al., 2022). For example, a study prepared five types of microcapsules with wall materials: waxy rice starch, modified waxy rice starch, modified starch composite maltodextrin, modified starch composite gelatin, and modified starch composite gum arabic. The microcapsules with a single-wall material showed the lowest encapsulation efficiency, storage stability, and antioxidant resistance. This was attributed to the inability to form a dense layer with single glutinous rice starch, leading to core material decomposition (Zhao et al., 2023). However, covalent chemical bonding and electrostatic interactions occurred when proteins and polysaccharides were mixed as wall materials (Lin et al., 2021). Proteins exhibit good emulsion stability and film-forming properties, while carbohydrates facilitate rapid vitreous membrane formation from proteins and polysaccharides, delaying oil diffusion through the wall material and enhancing the antioxidant protection of active compounds during long-term storage (Le Priol et al., 2022). Combining mixed wall materials can increase stability and emulsification. For example, adding other components to maltodextrin as a mixed wall material to encapsulate oil. The whey protein in the mixed wall material acts as an emulsifier, while Arabic gum offers excellent film-forming and emulsifying properties, and sodium alginate contributes biological adhesion, biocompatibility, and biodegradability. This combination forms strong hydrogen bonds with maltodextrin, resulting in a compact microcapsule structure that effectively protects the lipid from oxidation and air exposure, leading to a significant increase in stability (Yu et al., 2021). In a study, whey protein isolate (WPI), maltodextrin (MD), and sodium alginate were selected as wall materials for encapsulating ginger essential oil using a spray–drying method in a ratio of 1:3:0.01, leading to improved antioxidant properties. At 25 °C, the predicted shelf-life of mixed wall microcapsules was ∼1032 days longer than that of single-wall microcapsules (Hu et al., 2020). Moreover, the antioxidant properties obtained by using different encapsulation techniques for the same substance can vary significantly. For example, the oxidative stability of three spray–dried lipid encapsulation methods was compared with conventional spray–drying < anhydrous spray–drying < vacuum spray–drying. Conventional spray-dried particles exhibited lower oxidative stability than anhydrous spray-dried particles. Additionally, anhydrous spray–dried particles showed lower glass transition temperature, indicating a significant increase in sample deformation within a certain temperature range and relatively stable deformation in subsequent ranges. Additionally, they displayed reduced hygroscopicity, moisture content, and water activity compared to conventional spray-dried particles. Fish oil encapsulated in hydroxypropyl cellulose through conventional and anhydrous spray–drying demonstrated that anhydrous spray–drying, which uses methanol or ethanol instead of water, reduced water content and improved the oxidation stability of the fish oil (Zhu et al., 2021). Moreover, using nitrogen as a drying gas instead of air and lowering the intake temperature further reduced the lipid oxidation in anhydrous spray–drying (Encina et al., 2021). However, vacuum spray–drying was found to decrease the particle size and wall thickness, effectively reducing the contact between oxygen and oil droplets. The average diameter of vacuum spray-dried particles (6.9 µm) was significantly lower than that of spray–drying (14.6 μm). This facilitates the reduction of oxygen content in the particles, leading to higher oxidative stability of oils in vacuum spray-dried granules (Ramos et al., 2021). In addition to wall composition and encapsulation technology, several other factors have a significant impact on the antioxidant properties of lipids. These factors include the pH of the encapsulation system (Hu et al., 2020), the concentration of emulsion concentration and the size of the core particles. Careful consideration of these parameters is crucial to optimize the encapsulation process and enhance the stability and effectiveness of encapsulated lipids in various applications. However, the microencapsulation of oils and fats faces two main challenges. First, microcapsule products in powder form are susceptible to agglomeration during storage. This issue can be addressed by minimizing the adsorption of wall materials or adding anticoagulants into the formulation. Another challenge is the high equipment cost and energy consumption associated with this technology. It is Therefore, it is necessary to develop more cost-effective and energy-efficient encapsulation equipment to make this technology more accessible and sustainable.

#### 3.1.2 Bioactive components

**Fig. 1:**
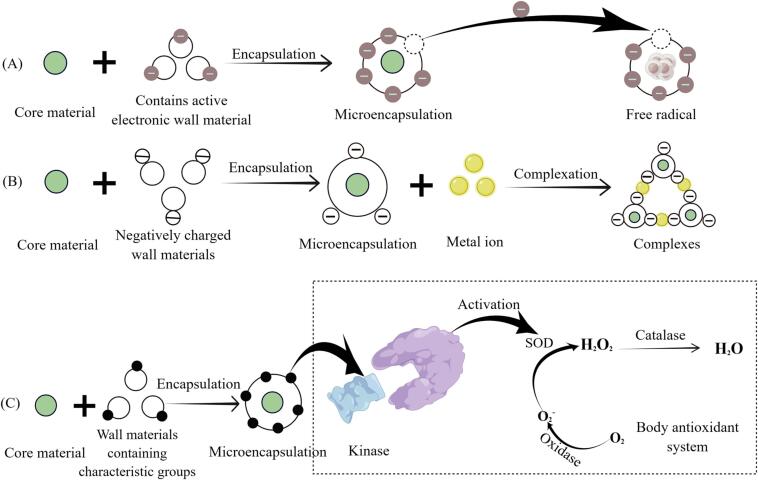
Antioxidant mechanism of microencapsulation of bioactive materials. (A) Direct scavenging free radicals; (B) Complexation with metal ions; (C) Activating the body's antioxidant system. (By Figdraw).

Bioactive components are substances found in trace or small amounts in organisms that play crucial roles in various life phenomena. These components include terpenoids, sterols, alkaloids, glycosides, resins, plant pigments, mineral elements, enzymes, and vitamins. Many of these substances are reductive or active in nature and are easily susceptible to oxidation. These substances are generally protected using direct scavenging free radicals, complexation with metal ions, and activating the body's antioxidant system (Fig. 1) (Filipsky et al., 2015). By means of encapsulation, some charged particles or ions in the wall materials react with free radicals, effectively protecting bioactive components from oxidation. For example, using the spray–drying method, curcumin can be encapsulated in soy protein isolate nanocomposites, soybean soluble polysaccharides, and maltodextrin. The wall material exhibits electrostatic adsorption, producing higher free radical absorption capacity. Nanomicroencapsulated curcumin demonstrates higher oxidative stability than free curcumin (Chen et al., 2020). Complexation of metal ions involves the formation of stable new ions by combining molecules or ions with metal ions or organic matter. For instance, citral microcapsules constructed with tannic acid–Fe III complex effectively protect the core material from oxidation, thereby preserving the physical and chemical properties of citral (Xu et al., 2022). Moreover, certain active ingredients, such as phenols and flavonoids, can activate the body's inherent antioxidant system, including superoxide dismutase, catalase, and other antioxidant factors, thus playing a significant antioxidant role. These active ingredients can serve as wall materials, acting as activators to enhance the antioxidant capacity of the encapsulated substances. For example, using a cross-coupling reaction of phenolic compounds and polymer self-assembly allows phenolic and aldehyde polymers to encapsulate probiotics at the nanoscale, forming a phenolic coating on the probiotic surface. The antioxidant properties of polymer-encapsulated probiotics contribute to their resistance to hydrogen peroxide- (H_2_O_2_) induced cellular oxidative stress. The encapsulated probiotic can scavenge approximately three times more oxygen free radicals than the original probiotics, resulting in 19 % higher bacterial activity than that of unencapsulated probiotics. The simultaneous encapsulation exhibits cytoprotective effects, enhancing the survival rate and adhesion ability of probiotics in an acidic gastric environment, thereby improving the delivery effect of probiotics and endowing them with antioxidant properties (Centurion et al., 2022). Additionally, phenolic substances reduce oxidative stress by activating the activity of superoxide dismutase and catalase. Among them, superoxide dismutase is also involved in scavenging superoxides that cause oxidative damage to cells, further reinforcing their beneficial antioxidant effects (Saha & Das, 2002). Microcapsule technology has demonstrated its ability to enhance the dissolution performance and stability of bioactive components while also providing protection against degradation and controlled release capabilities. Additionally, it is effective in masking the undesirable taste or flavors associated with various food-active compounds. For example, vitamins can be effectively treated using the oil-in-water emulsion embedding method. This not only enhances the utilization rate of vitamins but also ensures their effectiveness is maintained throughout storage. Consequently, microencapsulation provides a valuable commercial strategy for protecting vitamin the efficacy of vitamin encapsulation (Hu et al., 2022).

#### 3.1.3 Color protection

Color is a crucial sensory property of food, primarily determined by pigmented substances in the food components. Plants contain essential pigments like chlorophyll and carotenoids. Among these, carotenoids, in particular, are susceptible to discoloration due to their high reducibility, making them prone to oxidation during food production and processing. Encapsulation treatment offers a solution to enhance the antioxidant activity of pigmented substances, delaying their oxidation fading. The encapsulation treatment can modify the internal characteristics of particles, such as hydrophobicity, electrostatic forces, hydrogen bonding, and disulfide–thiol interactions, all of which contribute to improved oxidation stability (Chen et al., 2023). For instance, when anthocyanins are encapsulated, their 1,1-diphenyl-2-picrylhydrazyl (DPPH) free radical scavenging rate, 2,2′-Azino-bis(3-ethylbenzothiazoline-6-sulfonic acid) (ABTS) free radical, ferric ion reducing antioxidant power (FRAP), and copper-reducing antioxidant capacity decrease, leading to higher oxidation stability (Nguyen et al., 2022). The color protection benefits of encapsulation are not limited to plant pigments; animal-derived pigments can also be protected. For example, using chitosan and alginate microspheres as carriers for hemoglobin increases its stability during digestion, leading to a reduced release rate of hemoglobin, which results in a stable light brown color of the product (Silva et al., 2005). Plant pigments are widely used in production and daily life but are prone to oxidation and fading due to their instability. Encapsulation technology provides a solution by isolating oxygen and providing antioxidant functions. For example, the oxidative stability of mulberry anthocyanins can be improved using a double emulsion composed of pea protein isolate and xanthan gum (Aniya et al., 2023). Encapsulation of anthocyanins using double emulsion oil-in-water effectively isolates oxygen and prolongs the half-life of anthocyanin, ensuring stable pigmentation in liquid foods (Sebben et al., 2022). Additionally, encapsulation can delay fading caused by photooxidation. For example, lycopene can be encapsulated with cholesterol and ascorbic acid-6-palmitic acid as wall materials. After three months of storage in the dark, followed by five days of illumination, the color of encapsulated lycopene remains unchanged, while free lycopene exhibits noticeable fading (Luxsuwong et al., 2014). Pigments are functional components with wide application prospects owing to their safety, nutritional healthcare properties, and biological activity, attracting the attention of researchers and consumers alike. However, pigments are highly sensitive to environmental factors like light, pH, ultraviolet, temperature, oxygen, and heat. Encapsulation offers a viable pathway to add value to natural colors in functional foods, thereby expanding their benefits to a wider range of people.

### 3.2 Stability

#### 3.2.1 Protein activity

Proteins play a crucial role in food, serving not only as a vital nutrient but also contributing significantly to food quality (Yan et al., 2024). They influence various aspects of food processing and product characteristics. However, physical, chemical, and biological factors like oxidation and pH can alter the structure and function of proteins and their hydrolysates (peptides and amino acids) during food processing and storage. These alterations can lead to undesirable outcomes such as oxidation, denaturation, or degradation, resulting in nutrient loss, deteriorating quality, and reduced activity or function of the food (Zhang et al., 2022). Protein inactivation or denaturation can cause encapsulation treatment that not only improves the stability of the protein but also expands its application range. The preparation of microcapsules using covalent, hydrogen, and other bonds can enhance the oxidation stability of the capsules, allowing them to function effectively in a specific environment. For example, during gastrointestinal digestion, most small peptides in protein hydrolysates may be degraded into low-antioxidant activity peptides or amino acids, leading to decreased antioxidant activity (Mabrouk et al., 2021). However, after digestion, encapsulated protein hydrolysates have been found to exhibit free radical scavenging ability than their free state counterparts. Encapsulation can protect peptides from gastrointestinal secretions and enzymes due to the existence of ionic complexation and hydrogen bonding between phospholipids and peptides. For example, liposomal encapsulation protects antioxidant peptides in protein hydrolysates during storage and simulated digestion assays (Xu et al., 2021). Proteins are both hydrophilic and hydrophobic substances, and their structural stability largely relies on hydrophobic interactions within the molecules. In addition, there are various hydrogen bonds, salt bonds, van der Waals forces, and disulfide bonds within peptide chains and coordination bonds between peptide chains and contained metal elements (Chen et al., 2023). Therefore, common wall materials used in encapsulation include chitosan, β-cyclodextrin, sodium alginate, carboxymethylcellulose, proteins, and lipids. Protein activity can be influenced by environmental factors such as pH, ionic environment, and temperature. Strong acids or bases can break hydrogen bonds in proteins and form salts with free amino or carboxyl groups, leading to protein denaturation and inactivation (Xue et al., 2022). Encapsulation of proteins using acid and alkali-resistant wall materials helps protect proteins from destruction in strong acidic or alkaline environments. For example, using alginate as a wall material for protein encapsulation through the microgel method allows the protein to maintain a higher retention rate in an acidic environment, such as gastric acid (Zhang et al., 2016). Electrostatic repulsion plays a crucial role in maintaining protein conformation, but adding salt ions neutralizes the charge on the protein molecules, weakening or eliminating electrostatic repulsion and leading to protein aggregation or precipitation. Encapsulation treatment can prevent such aggregation and precipitation in the salt solution. For example, researchers used corn protein and whey protein nanoparticles as core materials and casein and alginate as wall materials to create biopolymer microgels through electrostatic complexation. The encapsulated protein remained stable in high salt solutions, resisting dissociation or aggregation. This function can transport proteins in condiments, sauces, and meat products with relatively high salt content (Zou et al., 2016). Proteins are also prone to denaturation by heat. However, encapsulated proteins can retain their activity even at higher temperatures, thus prolonging their storage period. For instance, egg white proteins in alginate microgels remain stable when heat-treated at 25 °C–35 °C. During storage, a portion of the protein is released rapidly in the first 60 min, followed by a slower release over time. At relatively high temperatures (65 °C–85 °C), proteins are released very slowly throughout the storage period, as egg white proteins form protein aggregates when heated at higher temperatures, reducing their diffusion through microgel pores and avoiding denaturation, thus extending the storage period (Su et al., 2019). Currently, protein embedding finds application in the processing of enzyme preparations. For example, maltodextrin has been used to microencapsulate pepsin and papain. This process yields a composite enzyme preparation with enhanced catalytic activity compared to pure enzymes. Furthermore, it helps maintain the protein hydrolyzing activity of the enzyme during storage. This microencapsulated enzyme preparation can be transformed into a direct-delivery product. It can also be used as an enzyme electrode for immobilized enzymes or biosensors through an immobilization process (Tikhonov et al., 2021).

#### 3.2.2 Physical stability

Food stability refers to how various environmental factors influence food during processing and storage, aiming to achieve acceptable stability under nonequilibrium conditions while maximizing desired food quality. The main factors affecting the stability of food are chemical and physical factors. Chemical factors, like oxidation resistance, have been discussed earlier, while physical factors include temperature, humidity, pressure, radiation (e.g., high-frequency electromagnetic field, microwave, ultraviolet, infrared, and radiation), noise, and vibration. Encapsulation technology plays a role in increasing the stability of the core material, preventing nutrient loss, and preserving sensory quality. For example, the thermal stability of curcumin decreases with increasing temperature. However, when encapsulated with carboxymethyl dextrin and zein, the active groups of curcumin are effectively protected in the hydrophobic cavity of these materials, significantly improving its thermal stability (Meng et al., 2021).In a microcapsule-based encapsulation system, the physical stability of the core material mainly depends on the strength of the bond between the wall and core materials. Adjusting the proportion of wall material components can enhance the chemical bond or intermolecular force between the wall and core materials, thus improving physical stability. For instance, when preparing β-carotene microcapsules using octenyl succinic anhydride (OSA) starch and trehalose as wall materials, a 3:7 ratio of OSA starch to trehalose leads to enhanced hydrogen bonding between β-carotene and OSA-starch, preventing β-carotene nanocrystal aggregation during spray–drying and significantly improving the physicochemical stability of the microencapsulated powder and aqueous dispersions (Zhang et al., 2021). In liquid encapsulation systems such as emulsions, physical stability also depends on the interaction within the system. Within a certain range, increasing the salt concentration in the emulsion shields against electrostatic repulsion between wall materials and reduces intermolecular spatial site resistance, resulting in increased stability. However, as the salt ion concentration continues to increase, the internal electrostatic repulsion between particles decreases, resulting in emulsion droplet aggregation and reduced stability. β-carotene is easily inactivated under light and heat conditions. One way to improve β-carotene stability is by preparing Pickering emulsion-embedded β-carotene using soy protein fiber polymer. Interestingly, when the salt ion concentration is lower than 600 mmol/L, the stability of β-carotene increases with the increase in salt ion concentration, resulting in a higher retention rate than the control group without salt ions (Tang et al., 2022). Light is another common physical factor that affects stability. Encapsulation treatment creates a physical barrier that isolates the core material from light, protecting its stability. For example, curcumin encapsulated in the cavity of γ-cyclodextrin–metal–organic framework crystals effectively prevents its activity from decreasing due to ultraviolet irradiation (Chen et al., 2021). Similarly, another study found that zein/carboxymethyl dextrin–curcumin nanoparticles exhibited better photostability than that of free curcumin (Meng et al., 2021).

### 3.3 Controlled release

#### 3.3.1 Sustained release

Sustained release is a technology that involves combining drugs or other bioactive substances with wall materials (usually polymer materials) to release them into the environment at a controlled rate over a specific period through diffusion. This concept is now applied in food production, environmental protection, and animal husbandry. Encapsulation treatment leads to two types of core material release: transient and slow release. Transient release occurs when external factors, such as changes in pH or enzymatic activity, lead to structural changes in the encapsulated material, resulting in its release. However, slow-release involves the degradation or dissolution of the wall material, gradually releasing the core material at a rate different from the initial release rate (Fathi et al., 2021). Various external environmental factors influence the release of bioactive compounds from the particles, such as pH values, ionic strength in the medium, temperature, magnetic field, and light radiation. At different pH values, the release of bioactive compounds from the particles may occur through different mechanisms such as disintegration, surface erosion, desorption, and diffusion (Zhao et al., 2019). For example, encapsulating ginger essential oil in chitosan particles minimizes the rate of ginger essential oil release in neutral media. However, the fastest release rate occurs in the most acidic medium (pH = 3). The increase in hydrogen ions at lower pH enhances the ion repulsive force of protonated free amino (NH_3_^+^), weakening the chitosan structure and allowing for further essential oil release. The viscous and digestive nature of the wall material can be used to control the time and rate of release (Karimi Sani et al., 2020). In the simulated gastrointestinal digestion test of microcapsules, the wall material containing amylose exhibited a better-sustained release effect than those without amylose. When gastric juice is digested for 100 min, the former releases less than 20 % of the core material, while the release of the latter reaches 50 %. The high viscosity of high amylose starch ensures the integrity of the microcapsule's wall, preventing core material release during gastric digestion. By contrast, the core material is released more rapidly in the small intestine due to pancreatic amylase decomposition and the acidic environment, achieving small intestine release (Yue et al., 2020). The hydrophilicity of the wall material, hydrogen bonds between the wall and core materials, and strong hydrophobic interaction also influence release. For example, the release rate of curcumin from zein/carboxymethyl dextrin nanoparticles greatly decreased after encapsulation, demonstrating an effective controlled release effect. This can be attributed to the presence of hydrogen bonds and strong hydrophobic interactions between curcumin and the delivery carrier. Additionally, carboxymethyl dextrin’s low solubility in low pH solutions protects the protein (zein) from enzymatic degradation in the gastrointestinal environment, achieving a slow disintegration and diffusion effect and effectively controlling the release of curcumin (Meng et al., 2021).

#### 3.3.2 Delivery

The delivery system is a reaction system that comprehensively regulates the distribution of substances in organisms in space, time, and dose. Its purpose is to ensure the precise delivery of the right amount of material to the right place at the right time, thereby increasing substance utilization efficiency, improving efficacy, and reducing costs. Delivery through encapsulation requires certain requirements for the type of wall material. Most food-related delivery processes occur in the acidic environment of the stomach and intestines, where they are exposed to digestive enzymes such as amylase, protease, and lipase in the digestive tract. Therefore, it is essential to select acid-resistant wall materials that can withstand digestive enzymes and allow delayed release and digestion of the embedded substance in the gastrointestinal tract. Nonstarch polysaccharides (chondroitin sulfate, pectin, chitosan, guar gum, and sodium alginate) are often used as wall materials for this purpose, as they can tolerate digestive enzymes and enable delayed release in the intestine (Xiao et al., 2022). Encapsulation also has the ability to change the hydrophilic/lipophilic, polar/nonpolar solubility, and other properties of the core material, which in turn affects its release location. For instance, when xanthan gum is mixed with starch as a wall material, the resulting microcapsules are less affected by enzymes. This slows down the erosion of the wall materials caused by digestive enzymes. The hydrophilicity and solubility of starch and xanthan gum can be used to encapsulate other insoluble substances, allowing them to be released into the gastrointestinal tract (Cai et al., 2019). This demonstrates the potential application of targeted delivery carriers for bioactive compounds (Wu et al., 2021). In a different study, zein and carboxymethyl dextrin nanoparticles served as effective curcumin delivery carriers. Among them, carboxymethyl dextrin, being hydrophilic, improved the solubility of microcapsules, while zein, a hydrophobic maize alcohol soluble protein, protected curcumin activity from degradation in the gastrointestinal environment. This shows that zein and carboxymethyl dextrin microcapsules can be used as effective delivery carriers for hydrophobic active compounds (Meng et al., 2021). Delivery systems find widespread use in drugs and health products. For example, probiotic encapsulation technology enhances the composition of the gut microbiota by safeguarding probiotic products from environmental factors and accurately delivering them to specific sites such as the colon. By controlling the dissolution rate and degree, probiotics can be delivered to the gastrointestinal tract with accurate targeting and effective colonization, making it the primary approach for intestinal probiotics (Kuo et al., 2022).

### 3.4 Microorganism

#### 3.4.1 Probiotics

**Fig. 2:**
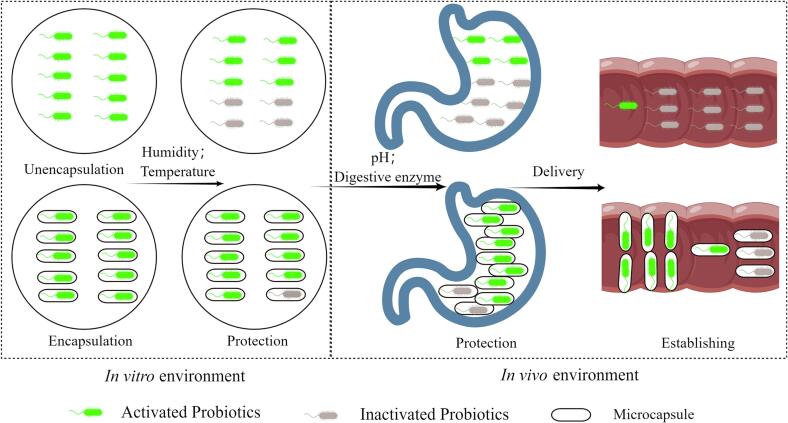
Schematic diagram for protection and colonization of probiotics using microencapsulation. (By Figdraw).

Probiotics are living microorganisms that provide health benefits to the host by improving the balance of gut microbiota. They are derived from human or animal normal flora, selected and artificially reproduced, and then formulated into live bacterial preparation through various methods and dosage forms to function effectively in the human body environment. Encapsulating probiotics requires ensuring their viability throughout the process. (Fig. 2) Encapsulation treatments have been shown to enhance the viability of probiotics (Azeem et al., 2023). Various wall materials are used for probiotic encapsulation, including polysaccharides (such as chitosan, sodium alginate, and resistant starch), proteins (like whey, protein, and gelatin), plant gums (such as gum arabic, carrageenan, pectin, and xanthan gum), and composite wall materials. The commonly used encapsulation methods include spray–drying, spray–freeze–drying, extrusion, air suspension fluidized bed, emulsification, and coagulation. Through encapsulation, the adverse effects of processing and storage conditions on probiotic viability, such as moisture content, moisture activity, temperature, and humidity, can be reduced (Barajas-Álvarez et al., 2023). For example, probiotics encapsulated with protein gel as a wall material exhibit better survival rates under conditions of high temperature, high pressure, shear stress, and gastrointestinal conditions (Mabrouk et al., 2021). Encapsulation also enhances probiotic survival at low moisture content and moisture activity. Under conditions of water activity of 0.25 and water content ranging from 4 % to 7 %, encapsulated probiotics can maintain their vitality, as the hydrophobic wall material protects the probiotics by binding to the hydrophobic structures on the cell membrane. For instance, the wall material adheres to the proteins of the cell membrane because of hydrophobic interactions, and probiotic cells are embedded in the wall of the capsule. This prevents the thermal inactivation of probiotics during the drying process (Luo et al., 2022). Additionally, appropriate wall materials used in encapsulation treatments confer probiotics with tolerance to the strong acid environment of the digestive tract and the presence of digestive enzymes. For instance, encapsulation protects the biological activity of probiotics in the harsh acidic environment of gastric juice (pH 2.0) until they reach the colon, thereby improving their bioavailability (Ajlouni et al., 2021). Under *in vitro* digestion conditions, encapsulated probiotics have demonstrated excellent activity and stability (Semyonov et al., 2012). For example, probiotics embedded in Pickering high internal phase emulsions, along with whey protein isolate and gallocatechin-3-gallate, form a conjugate through covalent bonding, effectively blocking the action of intestinal digestive enzymes and preserving the number and activity of probiotics (Qin, Gao et al., 2021). In probiotic products, encapsulation plays a vital role in enhancing the stability and viability of probiotic strains, thereby extending their shelf life. For example, probiotics can be encapsulated with alginate–gelatin through three-dimensional printing and freeze–drying, resulting in more than 106 CFU/g of equivalent probiotics remaining after 60 days of room temperature storage. This advancement opens up possibilities for customized strains and doses of active probiotics in shelf-stable, convenient casual foods or supplement products (Kuo et al., 2022). Another major application of probiotic encapsulation is to provide a controlled release for effective adhesion and colonization in the intestinal tract, ensuring targeted delivery (Reque & Brandelli, 2021). This involves overcoming two challenges: resisting the influence of gastrointestinal antibacterial factors and achieving the release of probiotics in the target environment. One such method involves encapsulating the liquid form of the Lactobacillus plantarum strain in a W1/O/W2 double emulsion internal water phase. The first layer provides resistance to gastric acid and pepsin, while the second layer provides resistance to bile salts and digestive enzymes. The W1/O/W2 double emulsion remains in a hydrogel state at pH levels below 4.0 and transforms into a solution state under neutral pH conditions, leading to the release of the core material due to Ca^2+^ dissociation with calcium alginate hydrogel. Therefore, the protection of W1/O/W2 double emulsion can successfully target the delivery of probiotics and ensure their release in the colon (Qin, Luo et al., 2021). Intestinal mucosal adhesion is crucial for achieving colon-targeted and efficient colonization. One method involves combining alginate and protamine through electrostatic droplets and layer-by-layer self-assembly, resulting in an enzyme-triggered fuse-like microcapsule. These multilayer microcapsules protect probiotics in the stomach and gradually decompose layer by layer under the catalysis of trypsin in the intestine, facilitating colonization. The microcapsules prepared using two layers of protamine-fused alginate protect probiotics from acid and bile salts, controlling cell release and promoting adhesion to the intestinal mucosa. leading to successful colonization in the proximal colon and distal ileum (Cheng et al., 2021). Microencapsulation is an effective approach to preserving the viability and functionality of probiotics under gastrointestinal conditions, such as storage capacity, survival ability, and function of microcapsule probiotics. However, the success of microcapsule probiotics depends greatly on the formulation and encapsulation process (Moghadam et al., 2021). Future work should focus on controlling the physical, chemical, and sensory changes of probiotic-rich foods to ensure the number and viability of probiotics during shelf-life and consumption.

#### 3.4.2 Antibacterial

Bacteriostatic agents play a significant role in food processing to inhibit bacterial growth. However, they have some limitations, such as low solubility, easy oxidation, poor sensory taste, and notably low thermal stability. Fortunately, the encapsulation treatment of these bacteriostatic agents offers a solution by improving their thermal and chemical stability, as well as enhancing their solubility under certain conditions. Additionally, encapsulation of bacteriostatic agents enables controlled release, leading to improved cell bioavailability and enhanced antipathogen efficiency (Deshmukh & Gaikwad, 2022). The application of encapsulation for bacteriostasis in food primarily focuses on essential oils, plant extracts, bacteriocins, and other bacteriostatic agents. Essential oils exert antibacterial activity through their phenols, alcohols, terpenoids, and aldehydes, which penetrate and destroy the cell membranes, causing cell content leakage and cell death (Liu et al., 2023). Essential oils exhibit broad-spectrum inhibitory effects and are naturally extracted, consistent with the growing market demand for health and safety (Teixeira et al., 2022). In addition to essential oils, natural plant components possess antioxidant, anti-inflammatory, and inhibitory activities, making them safe for food bacteriostasis. Although these components may have drawbacks, like unique taste and odors, microencapsulation effectively overcomes these shortcomings. For example, propolis exhibits antibacterial activity against Gram-positive (Lactobacillus monocytogenes and Staphylococcus aureus) and Gram-negative bacteria (Salmonella typhimurium and Escherichia coli O157:H7). However, its unique smell and slightly bitter and spicy taste limit its application in food. Through encapsulation with pea protein as a wall material and using spray–drying, the unpleasant taste and flavor of propolis can be masked, prolonging its antibacterial activity. This presents an application potential for propolis as a natural food preservative, inhibiting the growth of critical pathogenic bacteria (Jansen-Alves et al., 2019). Bacteriocins, which are substances produced by bacteria with bactericidal or bacteriostatic effects, typically consist of biologically active proteins or peptides. They exhibit a narrow activity inhibition spectrum against specific strains but are susceptible to environmental factors such as acidity, alkalinity, and high temperature. As a result, bacteriocins have a short duration of natural antibacterial activity in the environment, resulting in poor antimicrobial efficacy. Encapsulation treatment addresses this limitation by controlling the release of antibiotics, thereby prolonging their antibacterial activity and enhancing their effectiveness. For example, adding tannic acid to chitosan as a microcapsule wall material protects the protein of streptomyces lactis from oxidation. Additionally, tannins exhibit positive antimicrobial effects after forming complexes with Streptomyces lactis peptides (Leonida et al., 2023).

### 3.5 Flavor

#### 3.5.1 Taste substance

Taste is the feeling produced by the combination of taste substances and taste cell receptors. Encapsulation technology can achieve slow release of taste substances or mask undesirable taste. In particular, sweetness can be controlled through microcapsules after its release rate to achieve a lasting effect of sweetness. Gelatin and Arabic gum have been employed as key wall materials in this process. An illustrative case involves the encapsulation of aspartame using the double-emulsion complex coagulation method, with subsequent release in water at 36 °C and 80 °C. In both cases, a notable reduction in the release rate was observed, and higher temperatures did not lead to an increase in release. This shows that even under high-temperature conditions, encapsulation can also control the release of sweeteners (Rocha-Selmi et al., 2013). Salty taste primarily originates from low molecular inorganic salts. In low-salt foods, sodium chloride content is typically managed through sodium salt substitutes or the addition of alternative seasonings and nutrients. Encapsulation treatment can be used as one of the means of salt reduction. For instance, encapsulating sodium chloride with a hydrophobic wall material effectively reduces the release rate of sodium salt. This leads to a more sustained and stable release of sodium chloride in the mouth, thereby enhancing the perception of saltiness in the food product. Microcapsules were prepared through spray–drying using maltodextrin and octenyl succinic anhydride (OSA) modified starch as carriers for encapsulation sodium chloride. This approach significantly reduces the required amount of sodium salt while preserving the desired salty taste. Since maltodextrin-embedded samples release sodium at a faster rate than OSA-modified starch-embedded samples. The release rate of sodium can be adjusted by changing the proportion of wall material composition (Cai & Lee, 2020). Sour substances give a refreshing sensation, stimulate appetite, and can help reduce pH levels and inhibit the growth of spoilage bacteria. However, acidic substances can have an adverse effect on other components in the process, such as proteins and additives. To avoid such interactions, it is common practice to separate acidic substances from other raw materials until the final stages of production or add acidic substances post-encapsulation. For example, triglyceride and rosin ester have been used as the primary wall materials in the encapsulation of sour agent using the spray–drying method. This approach not only extends the shelf life of sour agent products but also prevents direct contact between the sour agent and acid-sensitive components during production (Chattopadhyay et al., 2023). Encapsulation technology also serves as a valuable tool for removing or masking unpleasant taste substances in food. For example, when bioactive peptides are used in food, encapsulation can be used as an effective means to reduce its unpalatable taste (Tran et al., 2023). Studies have shown that bitter peptides can be encapsulated in high internal phase emulsions in water-in-oil. These emulsions effectively shield the bitter peptides, which are readily soluble in the water phase, resulting in reduced bitterness and improved product stability (Gao et al., 2022).

#### 3.5.2 Odorant

Flavor plays a pivotal role in influencing product sales and consumer satisfaction. Volatile flavor substances trigger the physiological sensation of olfactory perception due to volatility. These flavor substances are predominantly organic, encompassing acids, alcohols, ketones, aldehydes, esters, and neutral compounds, as well as nitrogen and sulfur compounds and hydrocarbons (Andaleeb et al., 2023). These compounds have a low molecular weight (<400 Da) and, are very sensitive to heat, light, and oxygen, low boiling point, and high volatility. The production process, storage conditions, packaging materials, and the presence of other components in the product can all contribute to changes or even loss of flavor compounds. From an application perspective, encapsulation is commonly used to retain and control volatile aroma compounds, essential oils, and spices. Essential oils, being highly volatile, tend to quickly evaporate upon contact with air. Therefore, encapsulation methods are employed to microencapsulate essential oils and regulate the release of their volatile components. Interactions between volatile substances in essential oils and the wall material involve non-covalent interactions, such as hydrogen bonds, hydrophobic interactions, and van der Waals forces, which help slow the release of these volatile substances. For example, the sol–gel method has been used to prepare oil-in-water-in-oil (O/W/O) composite emulsions containing oil-core silica gel microcapsules. This method involves the assembly of SiO_2_ nanoparticles into shells in the aqueous phase to produce porous materials. The lipophilic essential oil is then filled with the internal oil phase of these nanoparticles, effectively controlling its release (Sousa et al., 2014). Encapsulation technology not only prevents the evaporation, oxidation, and moisture absorption of flavors and fragrances but also prolongs their shelf life and enables controlled release under predefined conditions. The release of volatile substances in spices can be well controlled by polar interaction and hydrogen bonding during encapsulation treatment of spices. For example, amphiphilic polymers are prepared in advance through polymerization to encapsulate perfume via spontaneous condensation in water. The condensed layer will not precipitate in solid form. Instead, it maintains suspension in the form of stable colloidal microcapsules to achieve sustained release of fragrances (Mamusa et al., 2021). When performing encapsulation of volatile aroma compounds, the primary aim is often to increase their solubility. Interaction between these volatile aroma components and the core material predominantly involves non-covalent bonds, such as hydrogen bonds, hydrophobic interactions, and van der Waals forces. Studies have shown that freeze–drying can effectively preserve truffle volatiles, while volatile aroma compounds wrapped by β-cyclodextrin can improve the volatile stability of truffle products, extending their storage time (Phong et al., 2022). Future research should focus on understanding the mechanisms governing flavor release and its applicability in various food and biological (gastrointestinal) systems, as well as the wall material characteristics and storage behavior of encapsulated powder.

## 4 New encapsulation technology

**Fig. 3:**
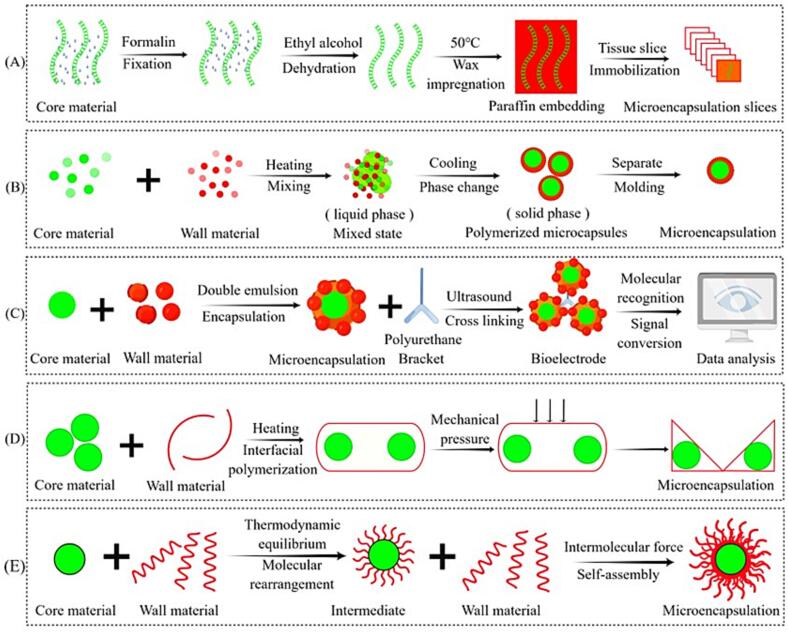
Mechanism of new encapsulation technology. (A) Formalin-fixed paraffin embedding method; (B) Phase change microcapsule; (C) Microcapsule biosensor; (D) Height difference nanoindentation; (E) Self-assembly technology. (By Figdraw).

Encapsulation technology has gained widespread applications in various fields, with many specific methods already well-developed in other industries. It is expected that this technology will be applied in the food industry in the future. Some of the promising encapsulation technologies include formalin-fixed and paraffin encapsulation methods (Alfieri et al., 2020), penicillinase immobilized phase change microcapsules (Liao et al., 2023), microcapsule biosensors (Nedellec et al., 2021), height difference nanoindentation (Jiang & Xie, 2019) and self-assembly technology (Jiang et al., 2023). Table 5 and Fig. 3 present the encapsulation technology expected to be applied in the food industry.

**Table 5:** Principle and application of new encapsulation technology.

Encapsulation System	Method/Principle	Current Application Field	Potential application in food industry
Formalin-fixed and paraffin encapsulation methods (Alfieri et al., 2020)	Tissues are placed in a certain concentration of formalin solution to induce cross-linking between nucleic acids and proteins. After fixation, the tissues are embedded in paraffin through dehydration, transparency, wax impregnation, and encapsulation.	Clinical medicine	Formalin-fixed and paraffin-embedded methods can accurately obtain high-quality and large quantities of ribonucleic acid in renal tissue. Furthermore, using biomarkers enables the precise detection of specific changes and their exact locations, allowing for targeted analysis.
Penicillinase immobilized phase change microcapsules (Liao et al., 2023)	Penicillinase is immobilized on chitosan coating through covalent bonding, resulting in a biocatalytic microcapsule system.	Microorganism	This technique can be used to study the intricate process of chemical changes and nutrient transfer in food. Penicillinase immobilized phase change microcapsules have promising applications in biosensing detection and biocatalytic removal of penicillin under microenvironment heat storage, temperature regulation, and thermal buffer conditions. They offer a more accurate and effective method for detecting and eliminating penicillin antibiotics in food, natural water systems, and industrial wastewater. Enzyme encapsulation plays a key role in this method and provides valuable insights for its application in catalysis within the food industry.
Microcapsule biosensors (Nedellec et al., 2021)	Porous microcapsules are formed through encapsulation with wall materials containing enzymes. These microcapsules can be converted into bioelectrodes using modified polyurethane scaffolds and used as biosensors. They enable the detection of substances and reversible inhibition.	Organism	Microencapsulation has revolutionized the application form of catechol-containing biosensors, presenting a versatile tool for detecting drugs, food contaminants, organic pesticides, and toxic heavy metals.
Height difference nanoindentation (Jiang & Xie, 2019)	This method involves interfacial polymerization between polymer and inorganic particles, reducing particle size through pressure. This method is better than nanoencapsulation technology.	Inorganic and nonmetallic materials	The height difference nanoindentation method enables nanoscale encapsulation in food through physical means, minimizing chemical changes and maximizing nutrient retention in food. Self-assembly technology utilizes the electrostatic interaction, hydrophobic interaction, and hydrogen bonding between the wall and core materials to make it spontaneously cross-linked to form an ordered structure.
Self-assembly technology (Jiang et al., 2023)	Structural units, such as molecules and nanoparticles, spontaneously form thermodynamically stable and well-organized aggregates through non-covalent bonding in various fields such as information, biology, and materials.	Information, biology, materials, etc.	Self-assembly encapsulation technology can be applied to biosensors, coatings, emulsions, and controlled release of active ingredients in the food industry.

## 5 Conclusions and outlook

Encapsulation technology offers a valuable solution for incorporating reactive and unstable substances, such as vitamins, proteins, or probiotics, into food matrices. It effectively enhances the stability and edible value of food, particularly in terms of functional components, nutrition, flavor, etc. As the demand for diverse and unique food products with added functionality increases, encapsulation technology has emerged as one of the most efficient methods to improve the stability, specificity, and bioavailability of key components in food products. Compared with other processing technologies, encapsulation technology can isolate the core material from the external environment. It can maintain the function or activity of key components in food to a greater extent. Furthermore, it can help in blocking some of the adverse ingredients. At the same time, by controlling the wall material digestion, the release rate of the core material can be controlled. Therefore, encapsulation technology can achieve various functions, including extending the shelf life of food, masking and reducing unwanted odor, and facilitating sustained release and delivery. Recently, significant progress has been made in developing new wall materials, technologies, and equipment for encapsulation. This has led to increased attention and application of encapsulation technology. However, challenges and limitations still exist, such as pollution and energy consumption during the encapsulation process, high preparation costs, limitations in the encapsulation rate, and precise controlled release. To address these problems, it is crucial to strengthen basic research in encapsulation technology. This involves actively exploring and developing new wall materials that are readily available, cost-effective, and versatile in their applications. Additionally, focus should be placed on developing new encapsulation technologies that are environmentally friendly, energy-efficient, and have low preparation requirements. With ongoing research and technological advancements, encapsulation technology is expected to become a conventional process in food processing. At the same time, the application of enzyme preparation and biosensor encapsulation in food processing and detection holds promising potential. Further research and exploration of new encapsulation technologies, such as self-assembly, will broaden the scope of applications for encapsulation technology. Ultimately, encapsulation technology will play a greater role in enhancing the quality and efficiency of the food industry, facilitating industrial upgrading.

## CRediT authorship contribution statement

**Yaguang Xu:** Writing – review & editing, Writing – original draft, Software, Investigation, Conceptualization. **Xinxin Yan:** Resources, Investigation. **Haibo Zheng:** Validation, Methodology, Funding acquisition. **Jingjun Li:** Validation, Investigation. **Xiaowei Wu:** Methodology, Investigation. **Jingjing Xu:** Validation, Investigation. **Zongyuan Zhen:** Writing – review & editing, Writing – original draft, Supervision, Resources, Project administration, Methodology, Investigation, Funding acquisition, Conceptualization. **Chuanlai Du:** Resources, Project administration, Funding acquisition.

## Declaration of competing interest

The authors declare that they have no known competing financial interests or personal relationships that could have appeared to influence the work reported in this paper.

## Data Availability

No data was used for the research described in the article.
